# Functional training for sarcopenia in older adults: mechanisms, benefits, and clinical implications

**DOI:** 10.3389/fpubh.2026.1829227

**Published:** 2026-07-17

**Authors:** Yueying Chen, Yingxu Pan

**Affiliations:** School of Physical Education and Training, Capital University of Physical Education and Sports, Haidian, Beijing, China

**Keywords:** activities of daily living, functional training, health promotion, older adults, physical function, sarcopenia

## Abstract

Sarcopenia in older adults has become a prominent public health challenge that jeopardizes their health and capacity for independent living. Characterized by the decline or loss of muscle mass, strength, and physical function, this condition leads to impaired activities of daily living and diminished quality of life in affected individuals. Conventional exercise interventions yield suboptimal improvements and suffer from low patient adherence. In contrast, functional training, which is designed to align with the daily living scenarios of older adults, can enhance their muscle strength, flexibility, coordination, and other physical capacities, thereby effectively ameliorating multiple physical functions in older adults with sarcopenia. This mini review synthesizes the mechanisms, benefits, and clinical implications of functional training across various physiological and functional systems in older adults with sarcopenia, and explores future research and development directions. It aims to provide a reference for the design and implementation of personalized and long-term intervention studies in this field.

## 1 Introduction

Sarcopenia is a progressive loss of muscle strength, mass, and function that mainly affects older adults (1). As the global population ages, this condition has become a major public health threat (2). Currently, the diagnosis of sarcopenia mainly relies on the standards proposed by the European Working Group on Sarcopenia in Older Adults (EWGSOP), the Asian Working Group on Sarcopenia (AWGS), and the International Working Group on Sarcopenia (IWGS), etc. There are certain differences among these standards in terms of the measurement methods and thresholds for muscle mass, muscle strength, and physical function. Epidemiological data indicate that sarcopenia affects 10–16% of older adults worldwide, significantly diminishing their quality of life (3). In China, the prevalence of sarcopenia among older adults is 20.7%, with the highest rate observed in those aged 80 years and above (45.4%), followed by individuals aged 70–79 years (27.2%) and 60–69 years (15.7%) (4). Sarcopenia is associated with multiple adverse health outcomes, including declined muscle function, cognitive impairment, and increased mortality (5). Traditional single-mode exercise interventions have shown limited efficacy in improving muscle mass and physical function, partly due to low adherence among older adults with sarcopenia (6). Functional training (FT), in contrast, enhances physical flexibility, coordination, and balance, while supporting gait control, postural stability, and performance of daily activities. These characteristics make FT more aligned with the real-life contexts of older adults (7). Several randomized trials show that FT boosts muscle strength, balance, and walking ability while cutting fall risk (8). These findings support FT as a safe and effective exercise-based intervention for the prevention and rehabilitation of sarcopenia in aging populations.

In this mini-review, we aim to systematically explore the potential mechanisms and application effects of functional training in older adults population with sarcopenia. It should be noted that currently, there are relatively few studies on the mechanisms of functional training for older adults sarcopenia, and the existing evidence still predominantly relies on traditional exercise studies, such as resistance training and aerobic training, rather than specific functional training. Although functional training and traditional training have partially overlapping mechanisms in improving sarcopenia, the direct evidence regarding functional training itself is still relatively preliminary. This review is based on clarifying this limitation, reasonably draw on the mechanism bases that have been proven effective in traditional training methods, and integrate relevant progress, in order to provide a reference for future research on the mechanism of functional training in older adults sarcopenia.

## 2 Method

### 2.1 Literature search strategy

To select the relevant literature for this small-scale review, a systematic search was conducted in four databases: Web of Science, Embase, PubMed, and Cochrane Library. The search period ended in March 2026. The following search terms were used: “functional training,” “functional exercise,” “functional performance,” “physical functional performance,” “functional movement,” “neuromuscular function,” “functional movement assessment,” “functional capacity evaluation,” “older adults,” “older adult,” “sarcopenia”. Studies of types such as randomized controlled trials, systematic reviews, Meta-analyses, and high-quality cohort studies were prioritized for inclusion, with no language restrictions on the publication. The compilation of search results will be managed using Endnote X9 software and conducted using a systematic approach, identifying and removing duplicates based on detailed information such as authors, titles, and publication dates.

### 2.2 Inclusion and exclusion criteria

#### 2.1.1 Inclusion criteria

(1) Study subjects: older adults with sarcopenia (≥60 years old) admitted to community or medical institutions; (2) Intervention measures: Clear “functional training” or exercise programs classified as functional training; (3) Control group: Non-functional training; (4) Outcome indicators: At least one assessment indicator of muscle mass, muscle strength, physical function, or daily living activity ability.

#### 2.2.2 Exclusion criteria

(1) Non-older adults individuals (age < 60 years) or non-sarcopenia patients; (2) Non-functional training intervention; (3) No reports of muscle mass, strength, physical function, or daily living activity; (4) Non-original research or unavailable full text.

### 2.3 Literature screening

**Figure 1:**
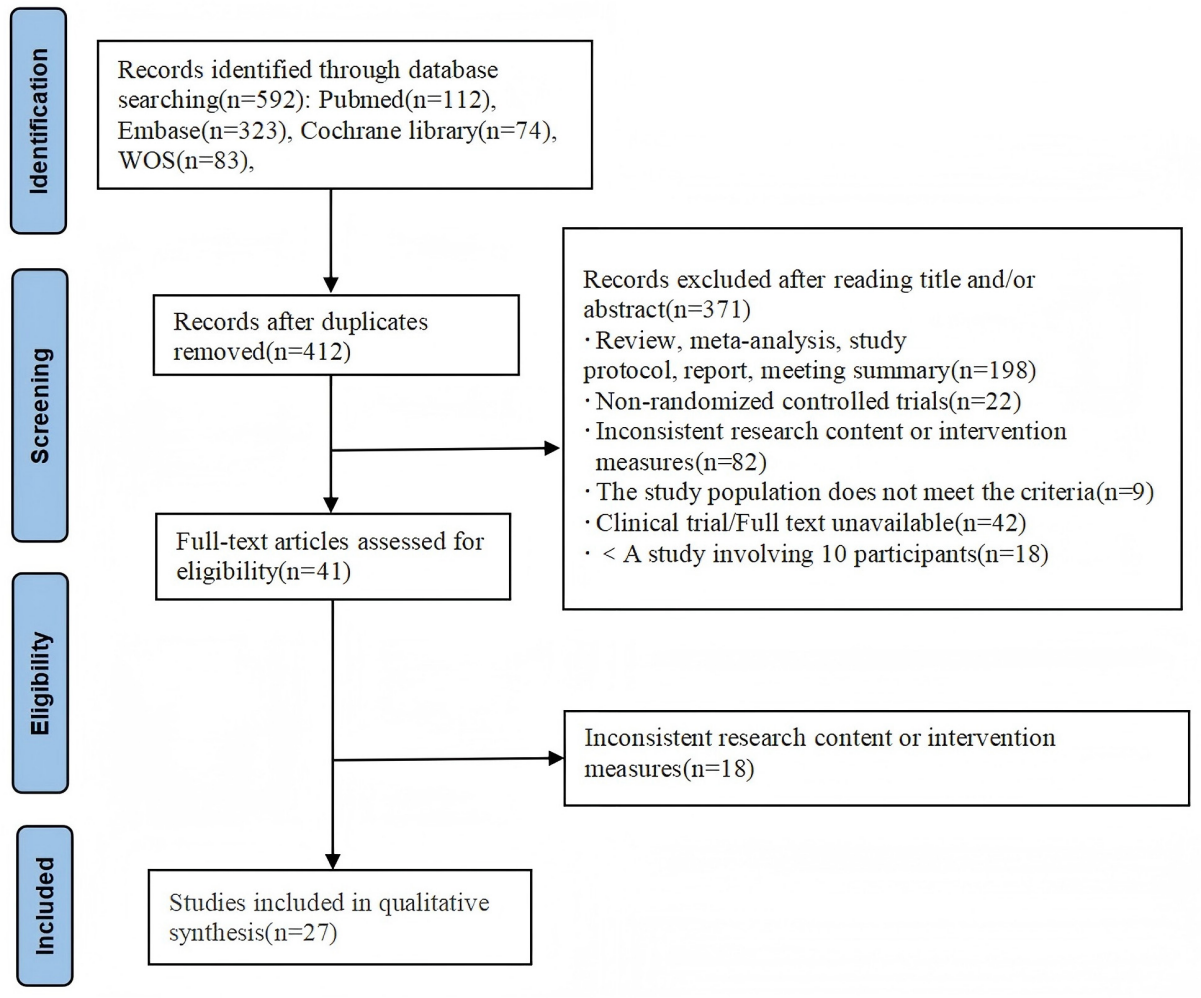
The literature screening process for studies on functional training in older adults with sarcopenia.

Two independent reviewers, Yueying Chen and Yingxu Pan, conducted a preliminary assessment of all the retrieved articles. In cases where the opinions of the two reviewers are inconsistent or there are differences, consultation with the third reviewer, Hai Wang, was conducted to make the final decision. Figure 1 shows the PRISMA flowchart for illustrating the screening process.

## 3 Evidence synthesis

### 3.1 Functional training overview

Functional training (FT) originated at the intersection of physical therapy and sports training, initially developed to restore daily function in rehabilitation and enhance sport-specific performance in athletes. As a goal-oriented intervention, FT aims to improve physical performance across exercise, rehabilitation, and daily life contexts. It emphasizes individualization and task specificity, with programs tailored to each person's functional needs (9). FT is now widely used in geriatric health promotion, improving muscle strength, muscle quality, balance, and quality of life in older adults (10). In this mini-review, functional training specifically refers to an exercise intervention model designed for older adults with sarcopenia, with the direct aim of enhancing their ability to perform daily activities. This training model is characterized by the coordinated participation of multiple joints and muscle groups. By simulating daily activities such as squatting, pushing, pulling, rotating, and walking, it improves various physical qualities such as strength, balance, coordination, and flexibility.

Based on the different intervention goals, the application of functional training in older adults sarcopenia can be classified into three categories. 1) Strength enhancement training. This training aims to delay the atrophy and loss of skeletal muscles, enhance basic strength, and provide core support for the body (11, 12). 2) Body control training. Centered on reducing the risk of falls, it improves balance, coordination, and flexibility to provide safety guarantees for patients with sarcopenia (13–15). 3) Daily activity simulation training. Simulating daily actions, it transfers the training effects to real life to maintain and restore the self-care ability of older adults with sarcopenia (16–18).

### 3.2 Potential mechanisms of functional training in older adults with sarcopenia

At present, there is limited research on the direct mechanism of functional training in relation to sarcopenia in older adults. However, a large number of studies on exercise-induced adaptations have provided a theoretical framework for understanding the possible mechanisms by which functional training may work. Therefore, in this section we have not only selected studies employing functional training protocols but also incorporated evidence from traditional exercise research, including studies on resistance training, aerobic exercise and multi-component training, to elucidate the potential mechanisms by which functional training can improve functional outcomes in older adults patients with sarcopenia.

#### 3.2.1 Restoring mitochondria and modulating inflammation

Older adults with sarcopenia face mitochondrial dynamic imbalance and oxidative stress dysregulation (19). Zhang et al. (20) summarized the research on sarcopenia and mitochondrial autophagy in the past 5 years, covering animal models, cellular models and human samples. They concluded that sarcopenia is closely related to mitochondrial dysfunction, and physical activity is a potent stimulus for promoting the generation of new mitochondria. Physical activity can promote mitochondrial biogenesis, optimize mitochondrial dynamics and enhance mitochondrial autophagy through the PGC-1α/NRF-1/TFAM pathway, thereby regulating the mitochondrial activity of older adults. Different types of physical activities can trigger mitochondrial autophagy in various organisms, thereby initiating and promoting this process. In terms of inflammation regulation, pro-inflammatory factors such as TNF-α and IL-6 exacerbate muscle atrophy and regenerative impairment in older adults sarcopenia patients by activating the NF-κB pathway, disrupting macrophage polarization, and inducing oxidative stress (21). A review on how exercise improves sarcopenia in older adults by regulating inflammatory pathways indicates that multi-component training (resistance + aerobic + balance Combined resistance, aerobic, and balance training) can effectively reduce the levels of systemic chronic inflammation by inhibiting the NF-κB and NLRP3 inflammasome pathways and reducing the release of pro-inflammatory factors such as TNF-α, IL-6, and CRP (22). Animal experiments further verified this mechanism. After 8 weeks of ladder climbing training for naturally aging mice, it was found that the training significantly reduced the mRNA levels of TNF-α, NF-κB, and IL-1β in skeletal muscle, promoted the polarization of macrophages to the anti-inflammatory M2 phenotype, and increased the cross-sectional area of muscle fibers. This indicates that ladder climbing training can alleviate sarcopenia in older adults by inhibiting the NF-κB signaling pathway and regulating macrophage polarization (23).

#### 3.2.2 Regulation of muscle anabolism and catabolism balance

Sarcopenia is marked by muscle cell loss, fibrosis, and fat infiltration, which reduce quality of life and increase mortality. Preserving muscle mass and strength is critical for maintaining physical function and wellbeing in older adults (24). Exercise promotes muscle protein synthesis and downregulates Atrogin-1 and MuRF-1 by activating the PI3K/Akt/mTOR pathway, thereby restoring the muscle protein homeostasis in older adults with sarcopenia (25). In terms of catabolism, the excessive activation of degradation pathways such as the ubiquitin-proteasome system, autophagy, and calpain in aged skeletal muscle is the core factor for the net loss of muscle mass. Endurance and resistance exercises counter this by inhibiting these pathways, reducing oxidative stress, and restoring the balance between protein synthesis and breakdown (26). In terms of anabolism, the core defect of sarcopenia in older adults lies in insufficient muscle protein synthesis and reduced activity of the synthesis signaling pathway. Daily stretching, strength training, etc., can induce the activation of the IGF-1/Akt/mTOR signaling pathway, thereby promoting muscle protein synthesis, improving synthesis resistance, and increasing muscle mass and strength (27). Moreover, multimodal training and bodyweight exercises and elastic band training can improve the quality and function of older adults skeletal muscle, delay the symptoms of sarcopenia, and delay muscle loss through activating the mTOR signaling pathway and upregulating the expression of muscle growth-related genes (28). It is worth noting that the rapid atrophy of older adults muscles due to synthetic resistance is characterized by a rapid loss of muscle mass equivalent to 2–3 years of normal aging. This highlights the central role of continuous exercise intervention in clinical practice (29, 30).

#### 3.2.3 Improving the muscle microenvironment and neuromuscular function

Age-related motor neuron degeneration may lead to a reduction in muscle fiber quantity and loss of skeletal muscle mass, serving as a significant cause of sarcopenia (31). One hallmark of sarcopenia is the loss of motor units and breakdown of the neuromuscular junction. Exercise addresses this by activating dormant satellite cells, lowering aging markers like p16INK4a, and promoting muscle stem cell repair. This helps maintain muscle mass and slow age-related muscle atrophy and strength decline (32, 33). The research has found that TRX suspension training reduces myostatin, increases follistatin, and improves neuromuscular control and neural-muscle junction structure, offering a safe way to enhance muscle and nerve function in older adults with sarcopenia (34). In addition, multi-component training (aerobic, strength, and balance training) can also effectively prevent the increase of serum CAF in older adults with sarcopenia, maintain the stability of the neural-muscle junction, and also improve muscle structure and physical function (35).

**Figure 2:**
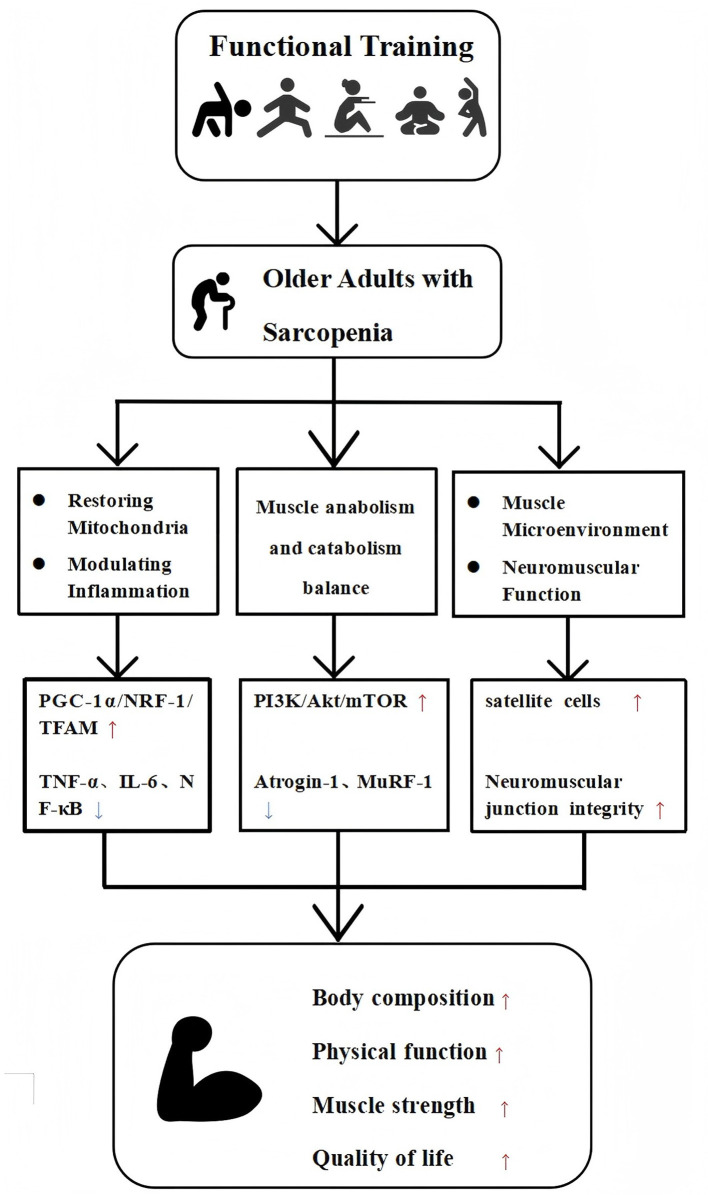
Mechanisms of functional training in older adults with sarcopenia.

In summary, functional training can significantly improve muscle cell energy metabolism, reduce chronic inflammation, regulate protein synthesis and degradation, enhance muscle mass and strength, and optimize neuromuscular transmission and motor unit recruitment in older adults patients with sarcopenia. These improvements contribute to enhanced muscle contraction and balance coordination, thereby improving daily activities such as walking and sitting. However, it should be noted that most current mechanistic evidence stems from broad exercise interventions—including resistance training, aerobic exercise, stretching, and multi-component training—rather than functional training itself. Although the PI3K/Akt/mTOR and NF-κB pathways have been well validated in animal and human studies involving conventional exercise regimens, there is insufficient direct evidence demonstrating their activation under specific functional training modalities. Additionally, many mechanistic studies rely heavily on animal models, and their applicability to human sarcopenia requires further validation. Consequently, current evidence regarding the efficacy of functional training in older adults sarcopenic patients remains limited, with most mechanistic insights derived from traditional exercise paradigms. Whether these pathways can be more effectively activated under specific functional training movements remains unclear and warrants further investigation. Figure 2 illustrates the mechanisms by which functional training benefits older adults patients with sarcopenia.

### 3.3 The intervention effects and research progress of functional training in older adults with sarcopenia

#### 3.3.1 Functional training based on muscle strengthening

Functional training has a positive impact on the muscle strength and quality of older adults with sarcopenia, especially in terms of body composition, muscle mass, and muscle strength (36). Functional training for sarcopenia combines resistance exercises with multi-joint, multi-plane movements to build muscle strength. Elastic band training is one effective example. In a 12-week trial of 40 older women with sarcopenia (three 60-min sessions per week), elastic band training improved lean mass, grip strength, leg strength, TUG time, and walking speed more than group dance (37). Similarly, in a randomized controlled trial involving 56 women (mean ± SD age 67.3 ± 5.1 years) with sarcopenic obesity, after 12 weeks (3 times a week, 55 min each time) of elastic band training, the experimental group showed sustained superiority over the control group in total body muscle mass, limb lean body weight, and skeletal muscle index at 3 and 9 months follow-up (38). Moreover, integrating two or more exercise programs in one training session is also a common and effective strategy to enhance the intervention effect of older adults sarcopenia. In a 12-week randomized controlled trial, 28 older women with sarcopenic obesity who performed cycling training (including standing, lunges, jumps, and backward kicks) showed increased lean mass, reduced fat mass and body fat percentage, and improved cardiovascular function (39). Similarly, in a randomized controlled trial of 61 nursing home residents with sarcopenia, the Otago exercise program improved muscle mass, strength, physical function, and quality of life compared to usual care (12). Liu Minjing et al.'s home-based resistance combined with aerobic walking study, and Kornanong YUENYONGCHAIWAT's walking combined with elastic band training study, both showed that these two intervention methods can improve muscle strength indicators such as knee extensor strength, grip strength, and walking speed in older adults with sarcopenia (40, 41). Besides the training program itself, multiple lifestyle interventions, such as nutritional supplementation, cognitive training, and changing training locations, may also affect the intervention effect of older adults sarcopenia (42). Chang et al. (43) demonstrated that resistance training combined with nutritional supplementation can improve muscle strength and muscle mass, and reduce inflammatory cytokine levels such as IL-1β and IL-6. The intervention location also has an impact on the intervention of older adults sarcopenia. Tsekoura et al. (44) in a randomized controlled trial of 54 older adults individuals with sarcopenia living in the community found that the group doing group exercise through the arrangement and connection of actions such as sitting, standing, and walking, outperformed home-based exercise in improving muscle mass, muscle strength, physical function, and quality of life, and the improvement effect was more lasting.

#### 3.3.2 Functional training based on neuromuscular control

Based on the data from the China Health and Aging Tracking Survey, sarcopenia is closely associated with the increased risk of daily living activities (Activities of Daily Living, ADLs) and instrumental daily living activities (Instrumental Activities of Daily Living, IADLs) impairments in older adults (45). Older adults with sarcopenia experience accelerated physical functional decline, an increased risk of falls, and a decline in independent living ability (46). Multiple studies have shown that incorporating exercises such as Tai Chi and daily movement skill practice into the functional training program for older adults sarcopenia patients has certain advantages in controlling neuromuscular responses, stabilizing postures, coordinating gait, and preventing falls. According to a randomized controlled trial, In a 12-week trial of 56 older adults aged 60–80, Tai Chi shortened lower limb muscle response time and reduced OSI, with correlated improvements indicating that Tai Chi lowers fall risk through better neuromuscular function (13). Similarly, in a randomized controlled trial involving 90 older adults with sarcopenia (average age 88.6 years, range 85–101 years), simplified eight-form Tai Chi training was also shown to improve multiple physical function indicators, static balance ability, and dynamic balance ability. This study also included whole-body vibration training as a control group, and the results showed that Tai Chi was significantly superior to whole-body vibration training in improving balance ability (47). In terms of body posture control and movement skill simulation training, through interactive walking, obstacle courses, carrying heavy objects, etc., movement skill exercises such as these simulate the action patterns in real-life scenarios, and combined with elastic bands, weighted bags, etc., to strengthen posture practice, can to some extent improve the body composition, muscle strength, gait parameters, and neuromuscular strategies of older adults sarcopenic obese individuals, thereby enhancing walking ability and functional capacity (48). Functional multi-component training based on the arrangement of daily life actions also has a good intervention effect. Using movements such as squats, sitting-to-standing, center-of-gravity transfer, stepping, balance control, etc., that are close to daily life, and conducting progressive resistance, balance, and gait multi-component training, can also improve the daily activity ability and physical function of older adults with sarcopenia (49).

#### 3.3.3 Functional training based on digital empowerment

Digital intervention refers to health promotion methods carried out by data-based tools such as mobile applications, wearable devices, and motion-sensing games. It can improve the physical functions of older adults in terms of sitting, standing, and walking, and enhance their physical activity levels and exercise compliance (50). It is particularly effective for individuals with physical disabilities, those living in rural and remote areas, and groups with limited mobility or difficulties (51). A randomized controlled trial involving 70 community-dwelling older adults with sarcopenia aged 60–75 years showed that AI remote training based on deep learning 3D human posture estimation technology has certain advantages in improving muscle mass (ASMI), 6-meter walking speed, TUG time, and quality of life (SF-36), and there is no significant difference in intervention effect between the AI remote training group and the traditional face-to-face training group. This can be used as a safe and effective alternative to traditional face-to-face rehabilitation training (52). Another randomized controlled trial of digital motion-sensing game training for older adults in long-term care institutions used the Nintendo Switch Ring Fit Adventure motion-sensing game for 12 weeks of sitting training. The results showed that the intervention group had improved frailty index, muscle mass, muscle strength, walking speed, daily activity ability, and cognitive function. Digital training with motion-sensing games can safely and effectively delay sarcopenia and improve the comprehensive health level of older adults in institutions. Moreover, digital functional training has the prominent advantage of high compliance (53). The application of digital empowerment methods in functional training for sarcopenia in older adults can break through geographical and transportation restrictions, and improve training effectiveness and participation through real-time action correction and game design, thus providing a new feasible path for home-based rehabilitation of sarcopenia. Table 1 shows the pathways and intervention effects of functional training in older adults with sarcopenia.

**Table 1:** Functional training in older adults with sarcopenia: characteristics and main outcomes.

References	Criteria for diagnosing sarcopenia	Total participants (*N*)	Group (*n*)	Intervention	Primary outcome measures	key findings
Pablo Valdés–Badilla et al. (37)	EWGSOP2	40	EBG:21, GBD:19	Elastic band training VS moderate–to–high intensity dance training	↑ fat–free mass, ↑ HGS (dominant and non–dominant), ↑ leg strength, ↓ TUG, ↑ gait speed	Elastic band training produces significantly greater responses on physical–functional performance regarding group–based dance in older women with sarcopenia
Chun–De Liao et al. (38)	SMI < 27.6%	56	EG:33, CG:23	Elastic band training VS Maintain daily activities	↑ muscle mass, ↑ muscle quality, ↑ functional mobility, ↓ TUG, ↑ gait speed	Elastic band training exerted a significant beneficial effect on muscle mass, muscle quality, and physical function in older women with sarcopenic obesity
Won–Sang Jung et al. (39)	AWGS 2019	28	EG:14, CG:14	Cyclic training VS Maintain daily activities	↓ blood pressure, ↑ vascular function, ↓ IL−6	The 12 week circuit exercise program improved vascular function, and decreased inflammatory cytokines in obese older women with sarcopenia
Pan N t al. (12)	AWGS 2019	61	EG:31, CG:30	Otago Exercise Program VS Maintain daily activities	↑SMI, ↑Skeletal Muscle Mass,↑ hand grip strength„↑gait speed, ↓Five–Times–Sit–to–Stand	12 weeks of Otago plus resistance training in pre–frail older adults with sarcopenia in nursing homes enhanced body composition physical function and quality of life
Minjing Liu et al. (40)	AWGS2019	86	EG:45, CG:41	Home–based functional training VS Maintain daily activities	↑ muscle strength, ↑ 6MWD, ↓ TUG	12 weeks of graded progressive home–based resistance combined with aerobic exercise in community–dwelling older adults with sarcopenia improved muscle strength balance flexibility and cardiorespiratory fitness.
Kornanong Yuenyongchaiwat et al. (41)	AWGS 2019	57	EG:28, CG:29	Walking–based functional training VS Maintain daily activities	↑ inspiratory muscle strength, ↑ functional capacity (6MWT), ↑ gait speed	A pedometer–based intervention program with TheraBand resistance exercise could improve cardio–respiratory performance and PA among older Thai individuals with sarcopenia
Ke-Vin Chang et al. (43)	EWGSOP	114	EG:57, CG:57	Lower limb functional training + nutrition VS No intervention	↑ grip strength, ↑ SMI, ↓ TNF–α, ↓ IL−1β, ↓ IL−6	In older adults with sarcopenia a 12–week intervention combining resistance exercise with nutritional supplementation reduced these pro–inflammatory cytokines while improving grip strength and skeletal muscle mass index
Maria Tsekoura et al. (44)	EWGSOP	54	SG:18, IHE:18, CG:18	Gait + balance + multi–joint functional exercises (group–based) VS Gait + balance + multi–joint functional exercises (home–based)VS Brochure only	↑ SMMI, ↑ HGS, ↑ knee strength, ↑ calf circumference, ↓ TUG, ↓ 4m walk time, ↑ QoL	Group–based exercise was more effective than home–based exercise in improving muscle mass, muscle strength, physical performance, and quality of life in older adults with sarcopenia
Dunbing Huang et al. (13)	AWGS	60	EG:30, CG:30	Simplified Eight–Form Tai Chi Chuan: Progressive Training VS Health education, maintain daily activities	↓ neuromuscular response time, ↑ postural control, ↓ fall risk	Twelve weeks of Tai Chi exercise significantly improved physical function, including neuromuscular response and dynamic postural control, in older adults with sarcopenia, thereby reducing their risk of falls
Yaqiong Zhu et al. 2019(47)	AWGS 2014	79	TC:24, WBV:28, CG:27	Simplified Eight–Form Tai Chi Chuan VS Whole–body vibration training VS Health education, maintain daily activities	↑ muscle strength, ↑ balance, ↑ gait speed, ↓ TUG, ↓Five–Times–Sit–to–Stand	In sarcopenic men of advanced old age, 8 weeks of Tai Chi or whole–body vibration exercise significantly improved lower limb muscle strength and physical function
Hamza Ferhi et al. (48)	handgrip force < 17 N, gait speed < 1.0 m/s, and BMI > 30 kg/m^2^	40	EG:20, CG:20	Motor skill training, posture training VS Maintain daily activities	↑ gait speed, ↑ step length, ↑ muscle strength, ↑ physical performance	A 24–week posture–strengthening–motricity program improved physical function (gait speed, step length, balance) and body composition in older adults with sarcopenic obesity
Hyuma Makizako et al. (49)	AWGS 2014	72	EG:36, CG:36	Balance + multi–joint functional training VS Maintain daily activities	↑ chair stand performance, ↓ TUG, ↑ gait speed	A 12–week multicomponent exercise program significantly improved physical function (chair stand and Timed Up–and–Go tests) in community–dwelling older adults with sarcopenia or pre–sarcopenia
Shichun He et al. (52)	AWGS 2019	75	TRHG:25, GTHG:25, AITHG:25	Taichi, in–person VS Taichi, remote + human supervision VS Taichi, remote + AI guidance	↑ ASMI, ↑ gait speed, ↓ TUG, ↑ QoL	For older adults with sarcopenia, AI–based remote training and traditional training are equally effective in improving muscle quality, physical function, and quality of life

EBG, Elastic Band Group; GBD, Group-Based Dance; EG, Experimental Group; CG, Control Group; SG, Supervised Group; IHE, Individualized Hhome-based Exercise; TC, Tai Chi; WBV, Whole-body Vibration; TRHG, Face-to-face Traditional Training Group; GTHG, General Remote Training Group; AITHG, AI-based Remote Training Group.

## 4 Discussion

Functional training (FT) is a safe and effective intervention for older adults with sarcopenia. By replicating daily activities such as walking, sitting, and standing, it holds significant clinical value for improving physical function and muscle strength (54). This training modality features high flexibility and good adaptability to individual needs; thus, its implementation for this population should be based on baseline muscle function and comorbidities, with the rational incorporation of resistance and aerobic exercises, strict intensity control, and dynamic regimen adjustments. This mini-review study indicates that functional training for older adults sarcopenia places greater emphasis on the coordinated participation of multiple joints and muscle groups, transferring training effects to real-life scenarios. For instance, Tai Chi, circuit training, and the Otago exercise. Additionally, digital functional training methods such as AI remote correction and sensory games have also demonstrated good training effects, providing a feasible alternative for home-based rehabilitation. Functional training is relatively flexible. When formulating training plans for older adults sarcopenia, it is necessary to comprehensively consider the baseline muscle levels and underlying diseases of older adults sarcopenia patients, reasonably combine daily action simulation training, control the exercise intensity, and dynamically optimize the plan.

The current research still has many limitations: Firstly, at present, the evidence base for strictly defined functional training for sarcopenia in older adults is limited. Although in this mini-review, the potential mechanisms, intervention mechanisms, and application progress of functional training in sarcopenia in older adults were analyzed. However, the current mechanistic evidence for functional training in older adults with sarcopenia is mostly inferred indirectly from traditional exercise studies. Direct confirmation through functional training-specific research is needed. Secondly, the current research sample sizes are small, and the age range of the samples, the severity of the disease, the intervention frequency, intensity, and cycle all have significant heterogeneity. The best dose-response relationship and long-term safety evidence are not yet sufficient; Thirdly, different studies respectively adopt diagnostic criteria such as EWGSOP or AWGS for sarcopenia, resulting in differences in the screening of research subjects, which may affect the objectivity and comparability of the results.

Future research should further investigate the personalized and long-term effects of functional training in older adults with sarcopenia, and develop tailored programs based on age, comorbidities, and physical function. Additionally, the integration of nutritional support and digital monitoring may help to define optimal nutritional protocols, key monitoring indicators, and standardized adjustment criteria. In conclusion, as a pivotal non-pharmacological intervention for older adults with sarcopenia, functional training requires continuous advancement in long-term efficacy and clinical accessibility to better improve their physical function and activities of daily living.

## References

[1] Nakade T Maeda D Matsue Y Kagiyama N Fujimoto Y Sunayama T . Prognostic value of sarcopenia definitions and outcomes consortium criteria in older patients with heart failure. JAMDA. (2025) 26:105350. doi: 10.1016/j.jamda.2024.105350 39542034

[2] Bing S Chen Z Wu D Yu B Qiu H Zhang Y . Evolution of sarcopenia status and risk of incident cardiovascular disease. Eur J Prev Cardiol. (2025) 27:zwaf115. doi: 10.1093/eurjpc/zwaf115 40036640

[3] Yuan S Larsson SC. Epidemiology of sarcopenia: Prevalence, risk factors, and consequences. Metabolism. (2023) 144:155533. doi: 10.1016/j.metabol.2023.155533 36907247

[4] Meng S He X Fu X Li Y Wang Y Zhang Z . The prevalence of sarcopenia and risk factors in the older adult in China: a systematic review and meta-analysis. Front Public Health. (2024) 12:1415398. doi: 10.3389/fpubh.2024.1415398 39161853 PMC11331796

[5] Zhuang M Gu Y Wang Z Liu Y Zhang H Chen X . Effects of 12-week whole-body vibration training versus resistance training in older people with sarcopenia. Sci Rep. (2025) 15:6981. doi: 10.1038/s41598-025-91644-2 40011687 PMC11865505

[6] Liu X Chen X Cui J. Therapeutic advances in sarcopenia management: From traditional interventions to personalized medicine. Clin Nutr. (2025) 51:187–97. doi: 10.1016/j.clnu.2025.06.007 40580805

[7] Zhang S Qian G Xu H Liu Y Wang P Chen Z . Effects of different exercise modalities on balance performance in healthy older adults: a systematic review and network meta-analysis of randomized controlled trials. BMC Geriatr. (2025) 25:570. doi: 10.1186/s12877-025-06212-0 40745584 PMC12315335

[8] Niyazi A Mir E Ghasemi Kahrizsangi N Hosseini S Rahmani A Mohammadi F . The effect of functional exercise program on physical functioning in older adults aged 60 years or more: A systematic review and meta-analysis of randomized controlled trials. Geriatr Nurs. (2024) 60:548–59. doi: 10.1016/j.gerinurse.2024.10.019 39461107

[9] Pantoja-Cardoso A Aragão-Santos JC Santos PJ Oliveira LM Silva RM Costa LF . Functional training and dual-task training improve the executive function of older women. Geriatrics. (2023) 8:83. doi: 10.3390/geriatrics8050083 37736883 PMC10514855

[10] Brogno B. Aging with strength: functional training to support independence and quality of life. Inquiry. (2025) 62:1–11. doi: 10.1177/00469580251348133 40575939 PMC12205185

[11] Han T Liang X Liu H Zhu M Shen S Song J . Muscle-building interventions improve glucose metabolism in elderly type 2 diabetic patients with sarcopenic obesity. Nutr Metab. (2025) 22:98. doi: 10.1186/s12986-025-00993-2 40804678 PMC12344927

[12] Pan N Chen Y Wang Z Liu C Tong J He Y„ et al. Effects of multicomponent otago exercise program with added resistance training on sarcopenia in pre-frailty older adults in nursing homes: a randomized controlled trial. Clin Interv Aging. (2025) 20:1927–43. doi: 10.2147/CIA.S552924 41246478 PMC12619544

[13] Huang D Ke X Jiang C Song W Feng J Zhou H . Effects of 12 weeks of Tai Chi on neuromuscular responses and postural control in older adults with sarcopenia: a randomized controlled trial. Front Neurol. (2023) 14:1167957. doi: 10.3389/fneur.2023.1167957 37188307 PMC10176447

[14] Zhou J Liu B Xu JF Wang Y Chen X Li H . Home-based strength and balance exercises for fall prevention among older individuals of advanced age: a randomized controlled single-blind study. Ann Med. (2025) 57:2459818. doi: 10.1080/07853890.2025.2459818 39918027 PMC11809163

[15] Alegre-Tamariz J Sánchez-Medina J Runzer-Colmenares FM Torres-Mallma C Vásquez-Rojas C Gutiérrez-Elías J . Impact of a functional gait training program as a complementary strategy to improve physical function in older adults: a randomized clinical trial. BMC Geriatr. (2025) 25:789. doi: 10.1186/s12877-025-06471-x 41120990 PMC12538938

[16] Duarte GP Ferraz DD Trippo KV Novais MCM Sales M Ribeiro NMDS . Effects of three physical exercise modalities on respiratory function of older adults with Parkinson's disease: a randomized clinical trial. J Bodyw Mov Ther. (2023) 36:425–31. doi: 10.1016/j.jbmt.2023.05.014 37949595

[17] de Rondão CA Mota MP Esteves D. Physical activity interventions in older adults with a cognitive impairment: A critical review of reviews. Aging Medicine. (2023) 6:290–306. doi: 10.1002/agm2.12256 37711255 PMC10498829

[18] Liu CJ Chang WP Shin YC Hu YL Morgan-Daniel J. Is functional training functional? a systematic review of its effects in community-dwelling older adults. Eur Rev Aging Phys Act. (2024) 21:32. doi: 10.1186/s11556-025-00369-8 39716049 PMC11664925

[19] Zhu Y Zhou X Zhu A Xiong S Xie J Bai Z. Advances in exercise to alleviate sarcopenia in older adults by improving mitochondrial dysfunction. Front Physiol. (2023) 14:1196426. doi: 10.3389/fphys.2023.1196426 37476691 PMC10355810

[20] Zhang X Liao S Huang L Wang J. Prospective Intervention Strategies Between Skeletal Muscle Health and Mitochondrial Changes During Aging. Adv Biol. (2025) 9:2400235. doi: 10.1002/adbi.202400235 39410835

[21] Antuña E Cachán-Vega C Bermejo-Millo JC Fernández-Martínez P de Toro-Martín J Bernal-López MR . Inflammaging: Implications in Sarcopenia. Int J Mol Sci. (2022) 23:15039. doi: 10.3390/ijms232315039 36499366 PMC9740553

[22] Liang Z Zhang L. Chronic inflammation as a driving factor for sarcopenia: an update on pathophysiology and future therapeutic targets. Front Pharmacol. (2026) 17:1733798. doi: 10.3389/fphar.2026.1733798 41808874 PMC12968189

[23] Cao Y Zhou J Quan H Zhang Y Wang L Li X . Resistance training alleviates muscle atrophy and muscle dysfunction by reducing inflammation and regulating compromised autophagy in aged skeletal muscle. Front Immunol. (2025) 16:1597222. doi: 10.3389/fimmu.2025.1597222 40529358 PMC12170331

[24] Carcelén-Fraile MdC Lorenzo-Nocino MF Afanador-Restrepo DF Moreno-Aznar LA Vicente-Rodríguez G Casajús JA . Effects of different intervention combined with resistance training on musculoskeletal health in older male adults with sarcopenia: a systematic review. Front Public Health. (2023) 10:1037464. doi: 10.3389/fpubh.2022.1037464 36684863 PMC9853907

[25] Yu M Ge L Fu C Zhang Y Wang X Li J . Unraveling the metabolic pathways between atherosclerosis and sarcopenia. Front Endocrinol. (2026) 16:1762825. doi: 10.3389/fendo.2025.1762825 41726101 PMC12920195

[26] Arif I Rasheed A Nazeer S Ahmad S Khan M Hussain A . Impact of physical activity and nutritional interventions on skeletal muscle atrophy. Eur J Transl Myol. (2025) 35:13177. doi: 10.4081/ejtm.2025.13177 40231413 PMC12265418

[27] Wang H He W Chen P Liu Y Zhang X Li Z . Exerkines and myokines in aging sarcopenia. Front Endocrinol. (2025) 16:1592491. doi: 10.3389/fendo.2025.1592491 40801027 PMC12339337

[28] Voulgaridou G Papadopoulou SD Spanoudaki M Chatzopoulou A Draganidis D Jamurtas AZ. Increasing muscle mass in elders through diet and exercise: a literature review of recent RCTs. Foods. (2023) 12:1218. doi: 10.3390/foods12061218 36981144 PMC10048759

[29] McKendry J Coletta G Nunes EA Holwerda AM Breen L Phillips SM. Mitigating disuse-induced skeletal muscle atrophy in ageing: resistance exercise as a critical countermeasure. Exp Physiol. (2024) 109:1650–62. doi: 10.1113/EP091937 39106083 PMC11442788

[30] Janssen TAH Lowisz CV Phillips SM Verdijk LB Snijders T. From molecular to physical function: the aging trajectory. Curr Res Physiol. (2025) 8:100138. doi: 10.1016/j.crphys.2024.100138 39811024 PMC11732118

[31] Wang HL Liu LL Tan ZY Zhang YQ Li XH Chen WJ . Research progress of sarcopenia: Diagnostic advancements, molecular mechanisms, and therapeutic strategies. Exp Mol Pathol. (2025) 143:104992. doi: 10.1016/j.yexmp.2025.104992 40815919

[32] Jackson MJ. Episodic denervation as a driver of loss of skeletal muscle redox homeostasis and muscle weakness in sarcopenia: Possible amelioration by exercise. Sports Medicine and Health Science. (2025) 7:341–50. doi: 10.1016/j.smhs.2025.02.002 40936663 PMC12421176

[33] Su Z Xiang L. Exercise, circadian rhythms, and muscle regeneration: a path to healthy aging. Front Neurosci. (2025) 19:1633835. doi: 10.3389/fnins.2025.1633835 41141425 PMC12546372

[34] Rezaei S Eslami R Tartibian B. The effects of TRX suspension training on sarcopenic biomarkers and functional abilities in elderlies with sarcopenia: a controlled clinical trial. BMC Sports Sci Med Rehabil. (2024) 16:58. doi: 10.1186/s13102-024-00849-x 38409184 PMC10898163

[35] Monti E Tagliaferri S Zampieri S Mosole S Pellegrino MA Doria A . Effects of a 2-year exercise training on neuromuscular system health in older individuals with low muscle function. J Cachexia Sarcopenia Muscle. (2023) 14:794–804. doi: 10.1002/jcsm.13173 36708273 PMC10067485

[36] Zhao H Cheng R Song G Zhang Y Wang X Liu H . The Effect of Resistance Training on the Rehabilitation of older adults with Sarcopenia: A Meta-Analysis. Int J Environ Res Public Health. (2022) 19:15491. doi: 10.3390/ijerph192315491 36497565 PMC9739568

[37] Valdés-Badilla P Guzmán-Muñoz E Hernandez-Martinez J Herrera-Valenzuela T Ramirez-Campillo R Alvarez C . Effectiveness of elastic band training and group-based dance on physical functional performance in older women with sarcopenia: a pilot study. BMC Public Health. (2023) 23:2113. doi: 10.1186/s12889-023-17014-7 37891589 PMC10604857

[38] Liao CD Tsauo JY Huang SW Ku JW Hsiao DJ Liou TH. Effects of elastic band exercise on lean mass and physical capacity in older women with sarcopenic obesity: a randomized controlled trial. Sci Rep. (2018) 8:2317. doi: 10.1038/s41598-018-20677-7 29396436 PMC5797161

[39] Jung WS Ahn H Kim SW Kim YJ Park HY Lim K. Effects of 12-week Circuit Exercise Intervention on Blood Pressure, Vascular Function, and Inflammatory Cytokines in Obese Older Women with Sarcopenia. Rev Cardiovasc Med. (2024) 25:185. doi: 10.31083/j.rcm2505185 39076488 PMC11267184

[40] Liu M Li J Xu J Chen Y Chien C Zhang H . Graded progressive home-based resistance combined with aerobic exercise in community-dwelling older adults with sarcopenia: a randomized controlled trial. Clin Interv Aging. (2024) 19:1581–95. doi: 10.2147/CIA.S473081 39355281 PMC11444075

[41] Yuenyongchaiwat K Akekawatchai C. Beneficial effects of walking-based home program for improving cardio-respiratory performance and physical activity in sarcopenic older people: a randomized controlled trial. Eur J Phys Rehabil Med. (2022) 58:838–44. doi: 10.23736/S1973-9087.22.07612-2 36416166 PMC10086760

[42] Lu Y Niti M Yap KB Tan CTY Nyunt MSZ Feng L . Effects of multi-domain lifestyle interventions on sarcopenia measures and blood biomarkers: secondary analysis of a randomized controlled trial of community-dwelling pre-frail and frail older adults. Aging. (2021) 13:9330–48. doi: 10.18632/aging.202705 33882026 PMC8064206

[43] Chang KV Wu WT Chen YH Chen LR Hsu WH Lin YL . Enhanced serum levels of tumor necrosis factor-α, interleukin-1β, and−6 in sarcopenia: alleviation through exercise and nutrition intervention. Aging. (2023) 15:13471–13485. doi: 10.18632/aging.205254 38032288 PMC10713395

[44] Tsekoura M Billis E Tsepis E Dimitriadis Z Matzaroglou C Tyllianakis M . The effects of group and home-based exercise programs in elderly with sarcopenia: a randomized controlled trial. J Clin Med. (2018) 7:480. doi: 10.3390/jcm7120480 30486262 PMC6306785

[45] Zhou H Ding X Luo M. The association between sarcopenia and functional disability in older adults. J Nutr Health Aging. (2024) 28:100016. doi: 10.1016/j.jnha.2023.100016 38267154 PMC12877767

[46] Gonçalves AK Griebler EM da Silva WA Sant Helena DP da Silva PC Possamai VD . Does a Multicomponent Exercise Program Improve Physical Fitness in Older Adults? Findings From a 5-Year Longitudinal Study. J Aging Phys Act. (2021) 29:1–8. doi: 10.1123/japa.2020-0070 33761457

[47] Zhu YQ Peng N Zhou M Liu PP Qi XL Wang N . Tai Chi and whole-body vibrating therapy in sarcopenic men in advanced old age: a clinical randomized controlled trial. Eur J Ageing. (2019) 16:273–82. doi: 10.1007/s10433-019-00498-x 31543722 PMC6728405

[48] Ferhi H Gaied Chortane S Durand S Beaune B Boyas S Maktouf W. Effects of Physical Activity Program on Body Composition, Physical Performance, and Neuromuscular Strategies during Walking in Older Adults with Sarcopenic Obesity: Randomized Controlled Trial. Healthcare. (2023) 11:2294. doi: 10.20944/preprints202306.0738.v1 37628492 PMC10454246

[49] Makizako H Nakai Y Tomioka K Taniguchi Y Sato N Wada A . Effects of a Multicomponent Exercise Program in Physical Function and Muscle Mass in Sarcopenic/Pre-Sarcopenic Adults. J Clin Med. (2020) 9:1386. doi: 10.3390/jcm9051386 32397192 PMC7291119

[50] Berry ECJ Sculthorpe NF Warner A Maden-Wilkinson TM Degens H O'Brien TD. A scoping review of the feasibility, usability, and efficacy of digital interventions in older adults concerning physical activity and/or exercise. Front Aging. (2025) 6:1516481. 40290578 10.3389/fragi.2025.1516481 PMC12021916

[51] Munce S. The Importance of Telerehabilitation and Future Directions for the Field. JMIR Rehabil Assist Technol. (2025) 12:e76153. doi: 10.2196/76153 40300168 PMC12076027

[52] He S Meng D Wei M Guo H Yang G Wang Z. Proposal and validation of a new approach in tele-rehabilitation with 3D human posture estimation: a randomized controlled trial in older individuals with sarcopenia. BMC Geriatr. (2024) 24:586. doi: 10.1186/s12877-024-05188-7 38977995 PMC11232209

[53] Tuan SH Chang LH Sun SF Li CH Chen GB Tsai YJ. Assessing the Clinical Effectiveness of an Exergame-Based Exercise Training Program Using Ring Fit Adventure to Prevent and Postpone Frailty and Sarcopenia Among Older Adults in Rural Long-Term Care Facilities: Randomized Controlled Trial. J Med Internet Res. (2024) 26:e59468. doi: 10.2196/59468 39024000 PMC11294767

[54] Sun P Yang J Li N Yang W Yang J Ran J . Effects of aquatic exercise compared with land-based exercise on the body composition and function of older adults with sarcopenia: protocol for a randomised controlled trial. BMJ Open. (2025) 15:e085474. doi: 10.1136/bmjopen-2024-085474 39819955 PMC11752027

